# Bmi deficiency causes oxidative stress and intervertebral disc degeneration which can be alleviated by antioxidant treatment

**DOI:** 10.1111/jcmm.15528

**Published:** 2020-06-24

**Authors:** Qunhu Zhang, Jie Li, You Li, Hui Che, Ying Chen, Jianghui Dong, Cory J. Xian, Dengshun Miao, Liping Wang, Yongxin Ren

**Affiliations:** ^1^ Department of Orthopedics First Affiliated Hospital of Nanjing Medical University Nanjing Jiangsu China; ^2^ Department of Orthopaedics Suqian First Hospital Suqian Jiangsu China; ^3^ Department of Orthopaedics Xuzhou Central Hospital, Xuzhou Clinical College of Nanjing Medical University, The Affiliated Xuzhou Hospital of Southeast University Xuzhou Jiangsu China; ^4^ Department of Hand Surgery Department of Plastic Reconstructive Surgery Ningbo No. 6 Hospital Ningbo Zhejiang China; ^5^ UniSA Clinical and Health Sciences and UniSA Cancer Research Institute University of South Australia Adelaide SA Australia; ^6^ State Key Laboratory of Reproductive Medicine The Research Center for Bone and Stem Cells Department of Anatomy, Histology and Embryology Nanjing Medical University Nanjing Jiangsu China

**Keywords:** Bmi‐1, cell apoptosis, intervertebral disc degeneration, N‐acetylcysteine, oxidative stress

## Abstract

The transcriptional repressor Bmi‐1 is involved in cell‐cycle regulation and cell senescence, the deficiency of which has been shown to cause oxidative stress. This study investigated whether Bmi‐1 deficiency plays a role in promoting disc degeneration and the effect of treatment with antioxidant N‐acetylcysteine (NAC) on intervertebral disc degeneration. Bmi‐1^−/−^ mice were treated with the antioxidant NAC, supplied in drinking water (Bmi‐1^−/−^+NAC). For in vitro experiments, mouse intervertebral discs were cultured under low oxygen tension and serum‐limiting conditions in the presence of tumour necrosis factor α and interleukin 1β in order to mimic degenerative insult. Disc metabolism parameters in these in vitro and in vivo studies were evaluated by histopathological, immunohistochemical and molecular methods. Bmi‐1^−/−^ mice showed lower collagen Ⅱ and aggrecan levels and higher collagen Ⅹ levels than wild‐type and Bmi‐1^−/−^+NAC mice. Bmi‐1^−/−^ mice showed significantly lower superoxide dismutase (SOD)‐1, SOD‐2, glutathione peroxidase (GPX)‐1 and GPX‐3 levels than their wild‐type littermates and Bmi‐1^−/−^+ NAC mice. Relative to Bmi‐1^−/−^ mice, the control and Bmi‐1^−/−^+NAC mice showed significantly lower p16, p21, and p53 levels. These results demonstrate that Bmi‐1 plays an important role in attenuating intervertebral disc degeneration in mice by inhibiting oxidative stress and cell apoptosis.

## 1 INTRODUCTION

Intervertebral disc degeneration (IDD) is associated with diverse aetiologies such as mechanical stress, injury, ageing, obesity and genetic factors.^1^, ^2^ Cellular senescence has been demonstrated to be a natural part of the disc ageing process. Furthermore, accumulated disc cell senescence has been identified to have a close association with enhanced catabolism, elevated inflammation and accelerated IDD.^3^ Intervertebral discs are composed of the nucleus pulposus (NP) and annulus fibrosus (AF). The extracellular matrix (ECM) contains aggrecan and collagen, which provide mechanical support.^4^ Disc degeneration causes a decrease in anabolism and increase in catabolism within the disc. Loss of water content and ECM breakdown are usually found in IDD,^5^ and IDD is also characterized by an increased expression of proinflammatory cytokines such as interleukin‐1 (IL‐1) and tumour necrosis factor α (TNF‐α).^6^, ^7^ The condition is also associated with ROS production and oxidative stress.^8^, ^9^ However, pathobiology for IDD is unclear.

B‐lymphoma Moloney murine leukaemia virus integration site 1 (Bmi‐1), a member of the polycomb group of transcriptional repressors, is involved in cell‐cycle regulation and cell senescence through inhibition of the p16^INK4a^/Rb and p19^AFR^/p53 pathways.^10^ A total‐body premature ageing phenotype is exhibited in Bmi‐1^−/−^ mice, including conditions such as severe neurological abnormalities, a generalized failure‐to‐thrive, osteoporosis, alterations in various haematopoietic cell lineages and a markedly shortened lifespan. Bmi‐1 protects against oxidative stress and apoptosis.^11^ In Bmi‐1^−/−^ mice, the increased level of reactive oxygen species (ROS) due to impaired mitochondrial function is sufficient to induce organism senescence by triggering DNA damage. Bmi‐1^−/−^ mice could be generally rescued by the treatment with the antioxidant N‐acetylcysteine (NAC), including a prolonged lifespan and an increased body size.^11^, ^12^ However, it is unclear whether Bmi‐1 deficiency could lead to IDD.

In this work, we explored the role of Bmi‐1 on mouse intervertebral disc degeneration using Bmi‐1 gene knockout mice in vivo and organ culture in vitro and investigated the effect of N‐acetylcysteine (NAC) treatment on intervertebral disc degeneration. This study has elucidated the association between Bmi‐1 and oxidative stress in IDD, which will provide a foundation for developing new drugs for attenuating IDD.

## 2 MATERIAL AND METHODS

### 2.1 Ethics

This work was implemented with the approval of the Ethics Committee of Nanjing Medical University. Animal use was approved by the Institutional Animal Care and Use Committee of Nanjing Medical University (approval number: IACUC‐1601253).

### 2.2 Animal groups

Bmi‐1 homozygote (Bmi‐1^−/−^) mice (129Ola/FVB/N hybrid background) and wild‐type (WT) littermates were generated and genotyped as described previously.^10^, ^13^ The mice were maintained in the Experimental Animal Center of Nanjing Medical University, Nanjing, China. Mice were raised on a standard pellet food and water at 24 ± 2°C and 40%‐60% relative humidity. The in vivo experiments employed 4‐week‐old male Bmi‐1^−/−^ and WT mice, which were divided into three groups: WT, Bmi‐1^−/−^ and Bmi‐1^−/−^+NAC groups. Mice in the Bmi‐1^−/−^+NAC group received NAC (1 mg/mL) in their drinking water after birth. Intervertebral discs, including the vertebral endplates, AF, and NP, were harvested after removing the soft tissues and posterior ligament. Six mice of each group were killed for further analysis.

### 2.3 Organ culture

Intervertebral discs of 4‐week‐old WT mice were rinsed in saline solution and placed in 6‐well culture plates. The specimens were then divided into the control, degeneration and NAC treatment groups and cultured in a hypoxia workstation. The discs in the degeneration group were cultured in Dulbecco's Modified Eagle Medium (SH30023.01, Hyclone, Uta, USA) containing 10% foetal bovine serum (10099141, Gibco, Australia), 10 ng/mL IL‐1β (DXT‐130‐101‐682, Miltenyi), 100 ng/mL TNF‐α, 50 μg/mL, L‐ascorbate (CYT‐252, ProSpec, Israel), 40 mmol/L NaCl and antibiotics (15140‐122, Gibco).^14^, ^15^ The discs in the NAC group were managed by the same medium but with NAC supplementation (2.5 mmol/L), while those in the control group were cultured in DMEM/F12 containing 10% FBS, 50 μg/mL L‐ascorbate, 40 mmol/L NaCl and antibiotics. In all groups, the medium was completely replaced every 2 days, and the discs were maintained for 7‐14 days.

### 2.4 Histochemical and immunohistochemical studies

Intervertebral discs were removed and fixed in a periodate‐lysine‐paraformaldehyde fixative overnight at 4°C. All specimens were decalcified in an ethylenediaminetetraacetic acid‐glycerol solution for 5‐7 days at 4°C. The decalcified discs were then dehydrated, embedded in paraffin and cut into 5‐μm sections by using a rotary microtome. The sections were stained with haematoxylin‐eosin (HE) and alcian blue (S19072, Yuanye) or was prepared for immunostaining. The intervertebral disc sections were fixed with cold acetone and incubated with rabbit polyclonal anti‐collagen II (1:400, ab34712, Abcam), rabbit polyclonal anti‐collagen X (1:200, ab58632, Abcam), rabbit anti‐matrix metalloproteinase 3 (MMP3) (1:200, ab52915, Abcam), rabbit polyclonal anti‐SOD‐1 (1:200, ab16831, Abcam) or rabbit anti‐p16 (1:200, ab211542, Abcam). Peroxidase‐conjugated goat anti‐rabbit IgG (1:1000, A9169, Sigma) was used as secondary antibody.

Slides were viewed with an Olympus CX31 microscope (Olympus). ImageJ (National Institutes of Health) was used to quantify the staining intensity. The integrated optical density (IOD) and area of interest (AOI) of all the positive staining were measured. The mean density (IOD/AOI) was then calculated. The total numbers of positive cells were counted in each image with 400 × magnification to analyse numbers of immune‐positive cells. The section areas of nucleus pulposus were calculated to analyse extracellular matrix. At least 3 sections are randomly selected for each sample, and at least three fields of view for each section were analysed. The mean and standard deviation of the mean (SD) were calculated for each group.

### 2.5 Western blot

Immunoblotting was carried out to examine protein expression of aggrecan, collagen II, collagen X, SOD1, SOD2, P16, P21, P53, Bmi‐1 and β‐actin in intervertebral discs. Total protein from intervertebral discs was extracted and quantitated using a lysis kit according to the manufacturer's protocol (Bio‐Rad). Equal amounts (30 μg) of protein were electrophoresed on 6%‐12% SDS‐polyacrylamide gels and were transferred to Immobilon P membranes (Merke Millipore). The membranes were blocked in 5% non‐fat dried milk in Tris‐buffered saline/Tween 20 (TBS‐T: 20 mmol/L Tris (pH 7.5), 150 mmol/L NaCl and 0.05% Tween 20) for 1 hour at RT. The following rabbit monoclonal or polyclonal antibodies were used: rabbit polyclonal anti‐collagen II (1:2000, ab34712, Abcam); rabbit polyclonal anti‐collagen X (1:1000, ab58632, Abcam); rabbit polyclonal anti‐aggrecan (1:2000, ab36861, Abcam); rabbit polyclonal anti‐Bmi1 (1:2000, ab38295, Abcam); rabbit polyclonal anti‐SOD1 (1:1500, ab16831, Abcam); rabbit polyclonal anti‐SOD2 (1:1500, NB100‐1992, Novus Biologicals, Colorado, USA); rabbit anti‐p16 (1:2000, ab211542, Abcam); rabbit anti‐p21 (1:2000, ab109199, Abcam); rabbit polyclonal anti‐p53 (1:2000, ab131442, Abcam); and rabbit polyclonal anti‐β‐actin (1:2000, ab8227, Abcam). Primary antibodies were incubated for 20 hours at 4°C with 5% non‐fat dried milk in TBS‐T. The membranes were then washed three times in TBS‐T and were incubated for 1 hour at RT with goat anti‐rabbit IgG (1:1000, 7074S, CST). Immunoreactive bands were analysed by Scion Image Beta 4.02 and visualized with ECL (Beyotime). The relative expression of each immunoreactive band was normalized to the expression of β‐actin.

### 2.6 Quantitative real‐time RT‐PCR

Total RNA was isolated from intervertebral disc by using TRIzol (Beyotime) in accordance with the manufacturer's instructions. PrimeScript RT Master Mix (Perfect Real Time, TaKaRa) was used to reverse transcribe RNA to cDNA. The expression levels of collagen II, aggrecan, SOD‐1, SOD‐2, Gpx1, Gpx3, p16, p21, p53, Bmi‐1 and glyceraldehyde‐3‐phosphate dehydrogenase (GAPDH, as the endogenous control) were detected by real‐time PCR using the cDNA samples (primer sequences and sizes of product are listed in Table 1). Relative mRNA expression levels were determined by the 2^−ΔΔCt^ method.

**TABLE 1:** Primer sequences of the genes for real time RT‐PCR

Gene name	Forward (5′>3′)	Reverse (5′>3′)
Collagen II	TGGACGATCAGGCGAAACC	GCTGCGGATGCTCTCAATCT
Collagen X	GATCATGGAGCTCACGGAAAA	CCGTTCGATTCCGCATTG
Aggrecan	GGTGAACCAGTTGTGTTGTC	CCGTCCTTTCCAGCAGTC
SOD‐1	GGTGAACCAGTTGTGTTGTC	CCGTCCTTTCCAGCAGTC
SOD‐2	CAGACCTGCCTTACGACTATGG	CTCGGTGGCGTTGAGATTGTT
GPX1	CAATCAGTTCGGACACCAGGAG	TCTCACCATTCACTTCGCACTTC
GPX3	CTTCTTCTTGTTGAGCTGGACTC	CTGTGGAGGTCACTGTAGACT
p16	GGCTTCACCAAACGCCCCGA	GGGAGAGGGTGGTGGGGTCC
p21	AGTATGCCGTCGTCTGTTCG	GACTGCAAGACAGCGACAAG
p53	GGTTCCTGCCCCAGGATGTTG	GGAACATCTCGAAGCGCTCA
Bmi‐1	GACCACTACTGAATATAAGG	CATTTGTCAGTCCATCTCTC
GAPDH	ACAACTTTGGTATCGTGGAAGG	GCCATCACGCCACAGTTTC

### 2.7 Statistical analysis

All results were represented as mean ± SE from three independent experiments. Statistical analyses were performed by the Student's *t* test and one‐way ANOVA in GraphPad Prism 5.0 (GraphPad Software, La Jolla, CA). A value of *P* < .05 was considered statistically significant.

## 3 RESULTS

### 3.1 *The mouse model of disc degeneration* in vitro

**FIGURE 1:**
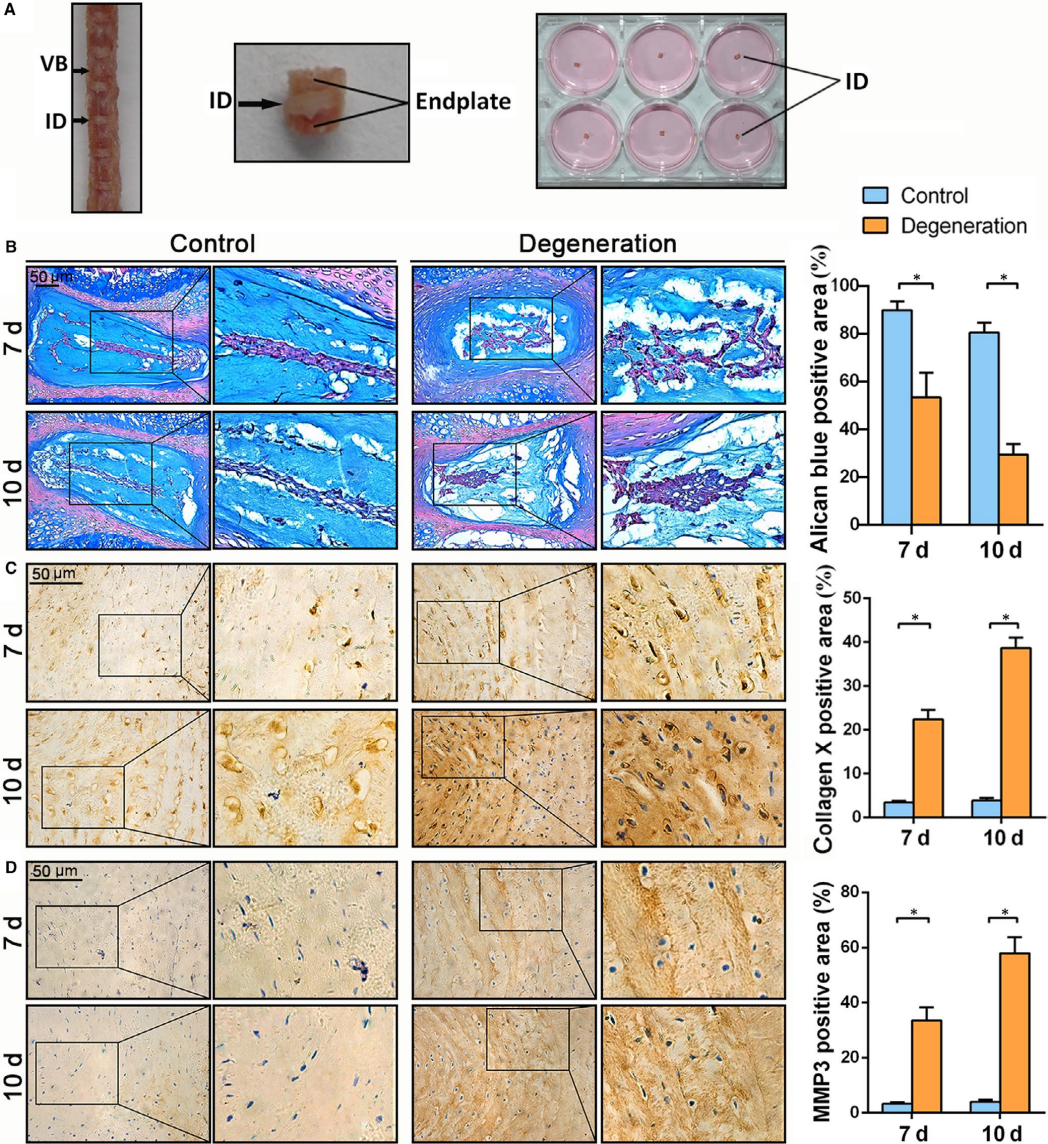
Alcian blue staining and collagen X and matrix metalloproteinase 3 (MMP3) immunostaining in mouse intervertebral discs cultured under the control or degeneration condition in vitro. A, Mouse intervertebral discs were removed under sterile conditions and cultured in 6‐well plate. B, The intervertebral disc structure and the expression of aggrecan in intervertebral disc were determined by HE‐Alcian blue composite staining. C, D, The expression of collagen X and MMP3 in intervertebral disc of mice was determined by immunohistochemistry. The positive areas of aggrecan, collagen X and MMP3 were analysed. (*P < .05)

Under sterile conditions, mouse intervertebral discs were removed with a portion of the endplate being retained. Using these discs, an in vitro disc degeneration model was developed by the intervention of IL‐1β and TNF‐α as described.^15^ The changes in the disc structure and aggrecan content were detected using HE‐Alcian blue composite staining, and changes in collagen X and MMP‐3 in the annulus fibrosus were analysed by immunohistochemistry. After 2 weeks of organ culture, the discs in the control group showed clearer borders of nucleus pulposus and annulus fibrosus than in degeneration group (Figure 1). Aggrecan was more abundant in the control group compared with the degeneration group. The discs of degeneration group contain more collagen X and MMP3 than those of the control group. With the prolonged culture time, the aggrecan contents in both groups gradually decreased, while those of collagen X and MMP‐3 in annulus fibrosus gradually increased. The above data suggest that an in vitro model of disc degeneration has been successfully established.

### 3.2 Bmi‐1 deficiency leads to IDD which can be alleviated by antioxidant NAC treatment

**FIGURE 2:**
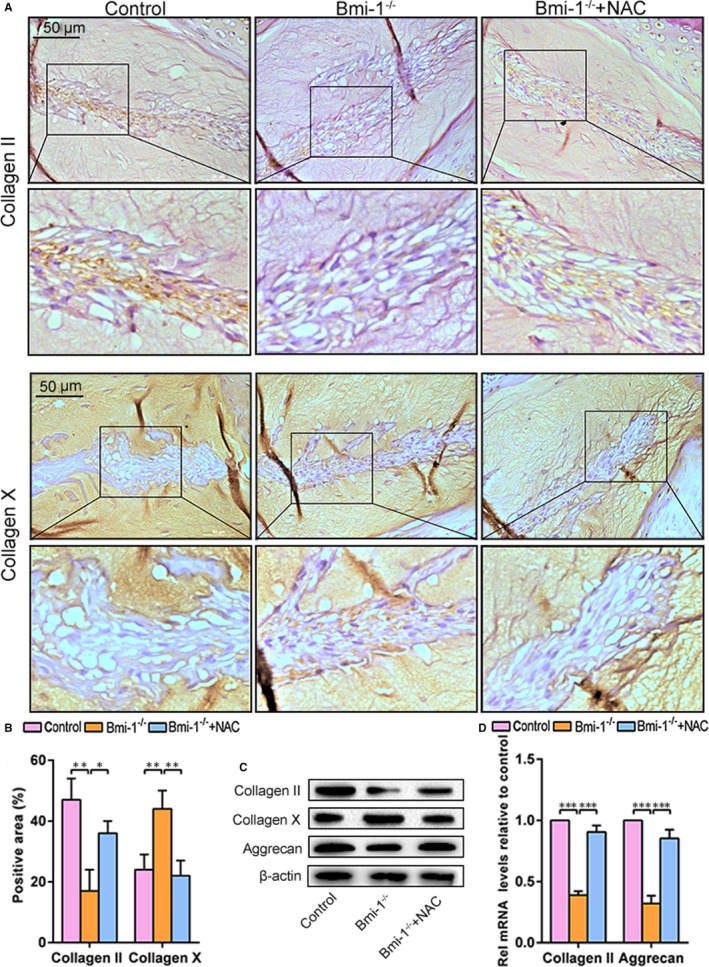
NAC treatment alleviates intervertebral disc degeneration caused by loss of Bmi‐1. A, Representative micrographs stained immunohistochemically for collagen II and collagen X. B, The percentage of areas with positive collagen II and collagen X staining. C, Western blots of intervertebral disc extracts showing levels of collagen II and collagen X. D, mRNA levels of collagen II and collagen X as determined by real‐time RT–PCR. Values are means ± SE of determinations in 6 mice of each group. * *P* < .05; ** *P* < .01; *** *P* < .001

To assess the effect of Bmi‐1 deficiency on IDD and potential rescuing effect by antioxidant NAC treatment, the intervertebral discs of 4‐week‐old mice of the WT, Bmi‐1^−/−^ and Bmi‐1^−/−^+NAC groups were analysed by immunohistochemical analysis, Western blot and real‐time RT‐PCR. Compared with WT and Bmi‐1^−/−^+NAC mice, fewer collagen II‐positive areas and greater collagen X‐positive areas were observed in Bmi‐1^−/−^ mice. Compared with Bmi‐1^−/−^ mice, significantly higher levels of chondrogenesis ECM proteins, including collagen II and aggrecan, and lower levels of cartilage hypertrophy protein collagen X were present in WT and Bmi‐1^−/−^+NAC mice (Figure 2A‐D). These data suggested that Bmi‐1 deficiency had promoted the degeneration of intervertebral discs and that the degeneration was alleviated to a great extent by NAC treatment.

### 3.3 Bmi‐1 deficiency causes oxidative stress in intervertebral discs which is rescued by NAC treatment

**FIGURE 3:**
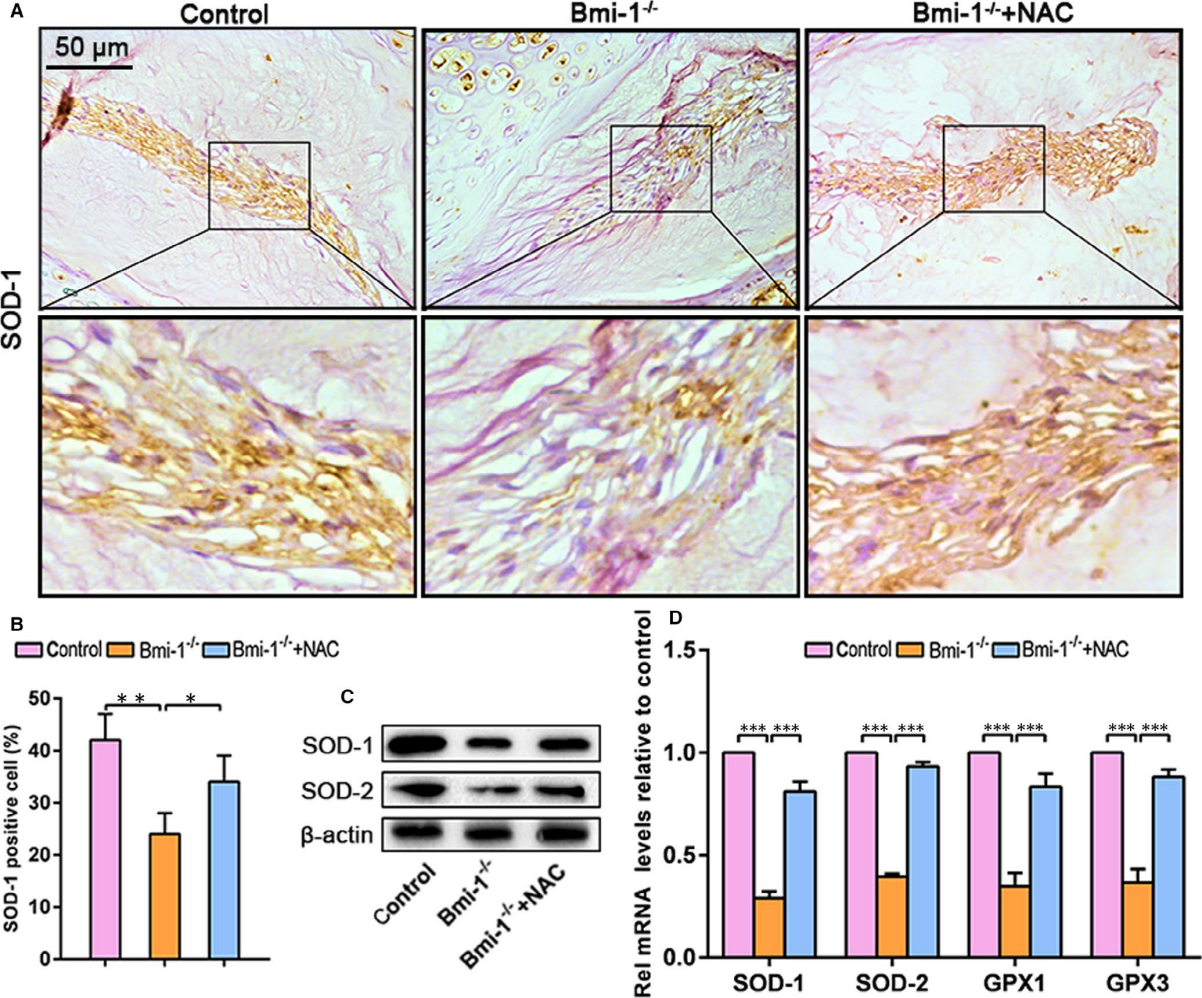
The loss of Bmi‐1 aggravates oxidative stress of intervertebral disc degeneration in mice, which can be alleviated by NAC treatment. A, Representative micrographs stained immunohistochemically for SOD‐1. B, The percentage of SOD‐1‐stained area. C, Western blots of intervertebral disc extracts showing levels of SOD‐1 and SOD‐2. D, mRNA levels of SOD‐1, SOD‐2, GPX1 and GPX3 as determined by real‐time RT–PCR. Values are means ± SE of determinations in 6 mice of each group. * *P* < .05; ** *P* < .01; *** *P* < .001

Compared with WT and Bmi‐1^−/−^+NAC mice, significantly fewer SOD‐1‐positive areas were exhibited in Bmi‐1^−/−^ mice (Figure 3A,B). As shown by Western blotting analyses (Figure 3C), expression levels of antioxidant proteins SOD‐1 and SOD‐2 were down‐regulated in Bmi‐1^−/−^ mice. The expression levels of antioxidant genes including SOD‐1, SOD‐2, glutathione peroxidase (GPX)‐1 and GPX‐3 were significantly higher in WT mice and Bmi‐1^−/−^+NAC mice than in Bmi‐1^−/−^ mice (Figure 3D). These data suggested that Bmi‐1 deficiency caused an increase in oxidative stress of intervertebral discs in mice, which can be alleviated by antioxidant NAC treatment.

### 3.4 Effects of Bmi‐1 deficiency and NAC treatment on cell cycle in intervertebral discs

**FIGURE 4:**
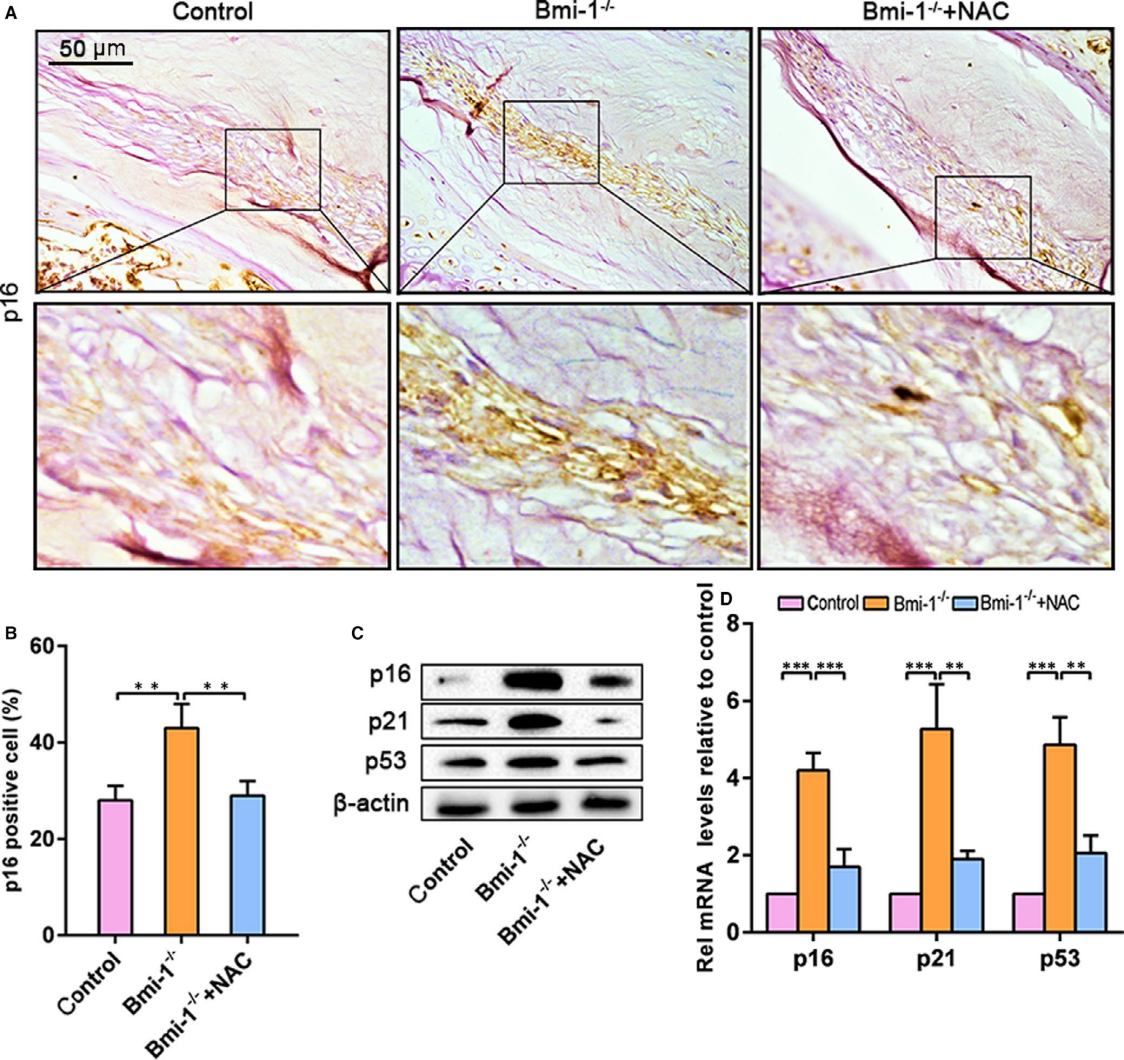
The loss of Bmi‐1 aggravates nucleus pulposus cell senescence in intervertebral disc degeneration in mice. A, Representative micrographs stained immunohistochemically for p16. B, The percentage of p16‐stained area. C, Western blots of intervertebral disc extracts showing p16, p21 and p53. D, mRNA levels of p16, p21 and p53 as determined by real‐time RT–PCR. Values are means ± SE of determinations in 6 mice of each group. *P < .05; **P < .01; ***P < .001

Bmi‐1 has been reported to be involved in cell‐cycle regulation and cell senescence by inhibiting p16^INK4a/Rb^ and p19^AFR^/p53 pathways.^10^ Therefore, we examined the expression levels of p16, p21 and p53. The immunohistochemical findings showed that p16‐positive areas were significantly greater in Bmi‐1^−/−^ mice than in WT and Bmi‐1^−/−^+NAC mice (Figure 4A,B). The expression levels of cell‐cycle proteins such as p16, p21 and p53 were dramatically up‐regulated in Bmi‐1^−/−^ mice relative to those in their WT littermates and Bmi‐1^−/−^+NAC mice (Figure 4C). Furthermore, expression levels of p16, p21 and p53 genes (as detected by RT‐PCR) were up‐regulated in Bmi‐1^−/−^ mice relative to those in WT and Bmi‐1^−/−^+NAC mice (Figure 4D). These results established that Bmi‐1 deficiency was associated with inhibition of cell proliferation and stimulation of cellular apoptosis in intervertebral discs of mice.

### 3.5 Organ culture for modelling IDD

**FIGURE 5:**
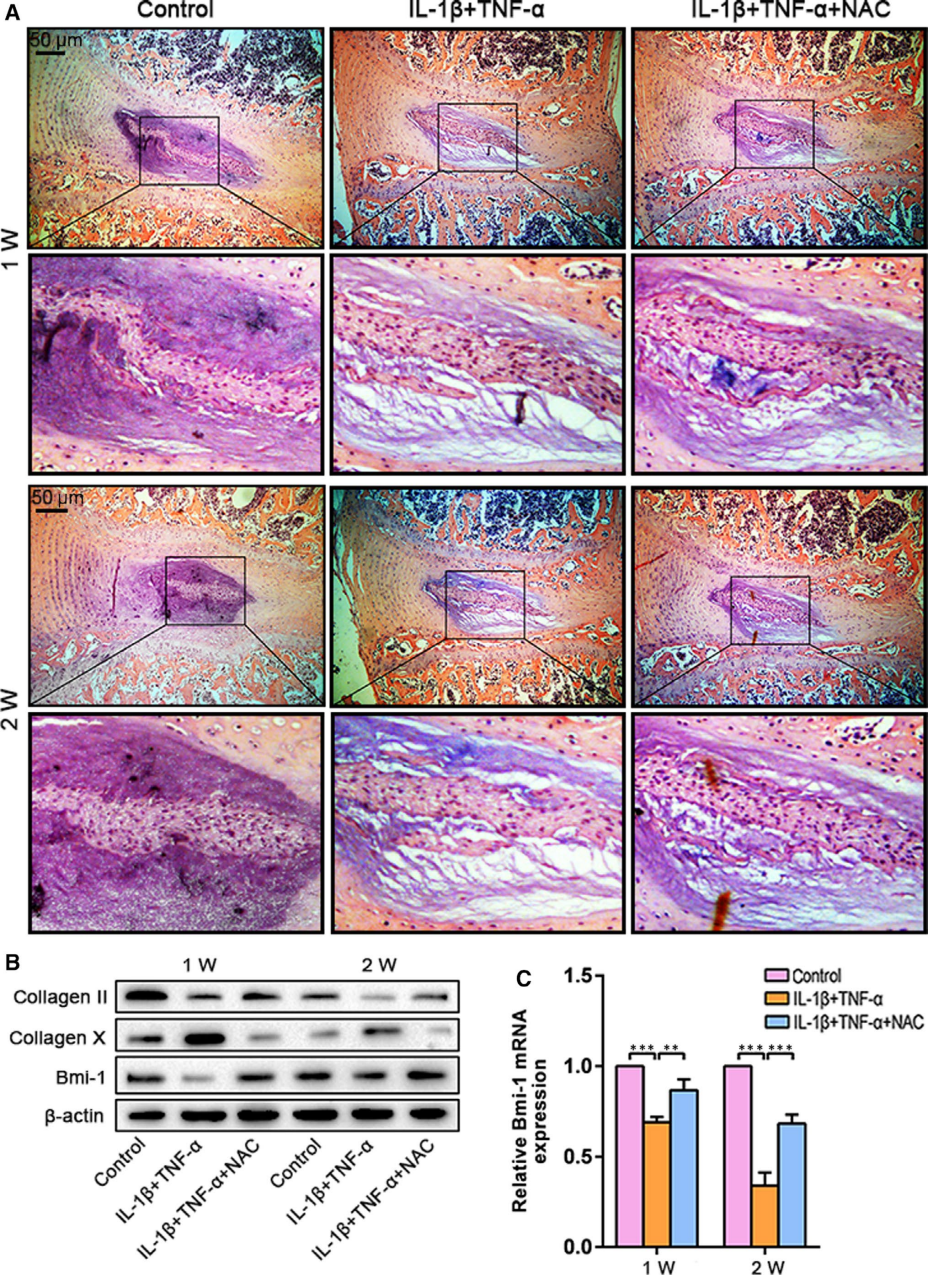
NAC alleviates nucleus pulposus cell degeneration induced by IL‐1β and TNF‐α. After one and two weeks of culturing, intervertebral discs were stained by haematoxylin‐eosin staining (A). The expression of collagen II, collagen X and Bmi‐1 was determined by Western blotting (B) and/or real‐time RT‐PCR (C). Values are means ± SE of determinations in 6 mice of each group. *P < .05; **P < .01; ***P < .001

The intervertebral discs of 4‐week‐old WT mice were cultured under various conditions with/without degenerative insult and NAC treatment (control, degeneration and NAC groups) and analysed by HE staining, Western blotting and real‐time RT‐PCR. Although some cells were undergoing necrosis, significant proportions of AF and NP cells were healthy until 2 weeks after treatment with NAC (Figure 5A). Relative to the control and NAC groups, decreased collagen II expression and increased collagen X expression were observed in the degeneration group. This result was consistent with that of the in vivo experiment (Figure 2A,B). Furthermore, the expression levels of the Bmi‐1 were decreased in the degeneration group and that NAC treatment could partially reverse this decrease (Figure 5B,C). These changes indicated that Bmi‐1 deficiency was associated with IDD in mice.

## 4 DISCUSSION

The aetiology and pathogenesis of IDD are still unclear. With natural ageing, cell senescence has deleterious impact on intervertebral discs causing IDD. It may play a major role in IDD as the senescent phenotype occurs at the right time.^16^ Senescence could be induced by increasing levels of ROS via DNA damage.^17^ In vivo, the active oxygen removal systems and the generation of reactive oxygen species (ROS) are in a state of dynamic balance.^18^ Excess oxidative stress, associating with free radical and reactive metabolite activities,^19^ affects health status and participates in the pathological process of diseases including IDD. Oxidative stress contributes to the cartilage endplate degeneration and NP cells apoptosis.^20^, ^21^, ^22^ Bmi‐1 has been shown to mediate oxidative stress response in various tissues.^17^, ^23^ Bmi‐1 protects dentin and mandible homeostasis and auditory hair cell survival by maintaining redox balance.^10^, ^17^ Besides, Bmi‐1 protects renal tubulointerstitial injury from oxidative stress after renal transplantation via mediating impaired mitochondria.^12^, ^23^ Here, a novel in vitro mouse model of disc degeneration has been successfully developed. We have investigated the role and mechanism of action of Bmi‐1 in IDD and found Bmi‐1 gene knock out caused IDD in the mutant mice compared with the WT, associating with the redox disbalance both in vivo and in vitro. In addition, we have demonstrated that the antioxidant NAC treatment can delay the disc degeneration caused by Bmi‐1 deficiency or IL‐1β and TNF‐α stimuli.

In this study, we developed a novel in vitro mouse model of disc degeneration under low oxygen tension and serum‐limiting conditions. Firstly, we successfully established an improved in vitro degeneration model of mouse discs using inflammatory factor intervention, which is an isolated model suitable for exploring the pathological mechanism of disc degeneration. According to previous research, the functional spine unit can only survive for 3‐5 days in the condition of a low concentration of foetal bovine serum medium for in vitro culture.^24^ Whereas, survival time can be prolonged for up to at least 10 days if 10% foetal bovine serum is present in culture medium.^25^, ^26^ In our experiment, intervertebral discs survived up to 14 days in 10% foetal bovine serum medium. Previous studies have established degeneration models using enzyme digestion or acupuncture methods.^27^, ^28^ However, this protocol does not really simulate real environment of intervertebral disc regarding the inflammation and metabolism, or the model construction method is complicated.^26^ In the current research, IL‐1β and TNF‐α increased the expression of MMP‐3 and collagen X and decreased the aggrecan level in the disc, a finding which resembles the trend in human degenerative discs.^29^, ^30^, ^31^, ^32^ Thus, our improved in vitro intervertebral disc degeneration model established with IL‐1β and TNF‐α intervention could be used in the further research for intervertebral disc degeneration.

In the disc tissues of our Bmi‐1^−/−^ mice, expression levels of oxidative stress response factors, including SOD‐1 SOD‐2 GPX‐1 and GPX‐3, were decreased. Additionally, important ECM components such as collagen II and aggrecan were decreased, while collagen X level was increased. In the healthy disc, large amounts of aggrecan and collagen II are contained. The internal pressure of the intervertebral disc, which provides a strong hydrodynamic system that fulfils the function of disc, is ensured by the adequate ECM. The catabolism of ECM and elevated levels of collagen X are known to be expressed in degenerated disc.^33^, ^34^, ^35^ Furthermore, up‐regulation of oxidative stress factors is found in degenerative discs.^20^, ^21^ Our result was consistent with the findings from these studies. Bmi‐1 deficiency caused IDD, and Bmi‐1 levels were decreased in an IDD model of mice. Bmi‐1 was also decreased in our IL‐1β and TNF‐α‐induced degeneration model in vitro. Thus, the potential mechanism underlying IDD caused by Bmi‐1 deficiency results from the increased oxidative stress.

Furthermore, Bmi‐1 also regulates cell‐cycle regulation and cell senescence through the inhibition of the p16 ^Ink4a^ and p19 ^Arf^/p53 pathways, and Bmi‐1 has been shown to play a critical role in maintaining the self‐renewing of stem cells in various many tissues, such as bone marrow mesenchymal stem cells, hematopoietic stem cells, lung stem cells, neural stem cell and intestinal stem cells.^10^, ^36^, ^37^ Bmi‐1 also prevents senescence by maintaining the mitochondrial function and redox balance.^23^ Bmi‐1 inhibits the expression of tumour‐suppressor genes, including p16, p19 and p27, to maintain the self‐renewal of bone marrow mesenchymal stem cells. The absence of Bmi1 involves in mitochondrial function and ROS homeostasis by increasing expression of a collection of gene products. Bmi‐1 is essential for the proliferation and survival of self‐renewing adult hematopoietic stem cells by downstream effectors of Bmi‐1, including p16^Ink4a^, p19^Arf^ and p53. Our results in intervertebral discs were similar to these tissues. Here, the expression levels of p16, p21 and p53 proteins were significantly up‐regulated in Bmi‐1‐deficient mice compared with the WT, suggesting that the premature IDD caused by Bmi‐1 deficiency in mice is related to the deficient inhibition of p16 and p53 signalling pathways.

NAC is an essential precursor to many endogenous antioxidants, which are involved in the decomposition of peroxides and attenuation of oxidative stress by replenishing intracellular glutathione stores.^38^ In clinical trials, NAC has been shown to improve lung function in patients with chronic obstructive pulmonary disease, which highlights its potential benefit in ROS‐directed therapy.^39^ Results of the current study demonstrated that IDD caused by Bmi‐1 deficiency was rescued to a great extent by NAC treatment in mice, which was accompanied by decreased resultant oxidative stress.

**FIGURE 6:**
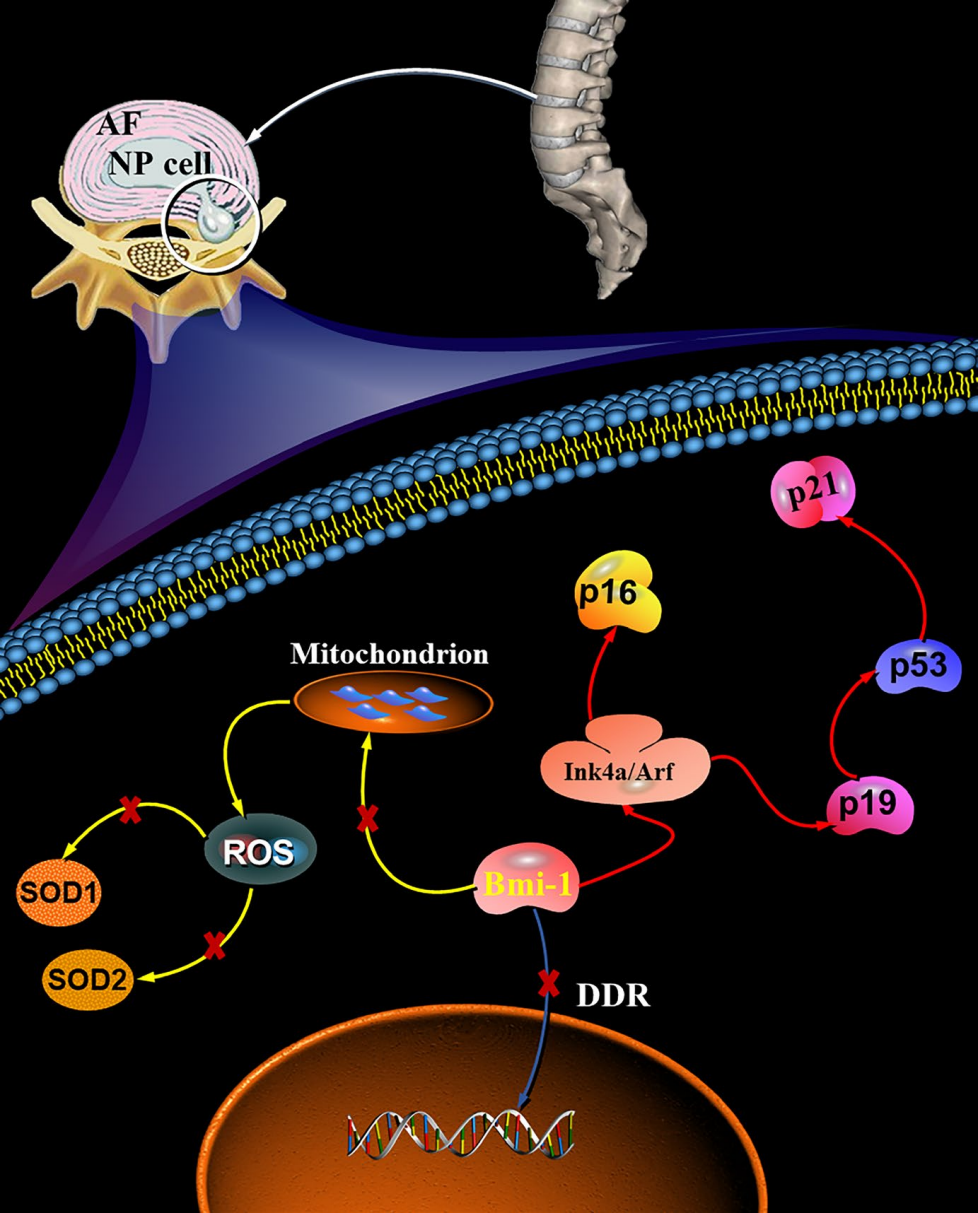
Proposed model depicting the mechanism of Bmi‐1 in regulating oxidative stress in IDD

Taken together, from the data in the current study, a model illustrating the possible mechanism of Bmi‐1 regulating oxidative stress response upon intervertebral disc degeneration can be proposed (Figure 6). Bmi‐1 deficiency directly activates the p16 and p19 signalling pathways by increasing the expression of p16, p21 and p53, which regulates cell‐cycle and cell senescence. An increased oxidative stress level that induced by Bmi‐1 deficiency results in the decrease of SOD‐1 and SOD‐2. Subsequently, Bmi‐1 deficiency contributes to the loss of ECM components, such as aggrecan and collagen II. We speculate that molecular studies of the Bmi‐1 deficiency may provide new solutions for preventing the degeneration of intervertebral discs. In conclusion, our study demonstrates that IDD caused by Bmi‐1 deficiency is associated with increased oxidative stress and that NAC is an effective option for the treatment of IDD.

## CONFLICT OF INTEREST

The authors confirm that there are no conflicts of interest.

## AUTHOR CONTRIBUTION

**Qunhu Zhang:** Data curation (equal); Formal analysis (equal); Writing‐original draft (equal); Writing‐review & editing (equal). **Jie Li:** Data curation (equal); Formal analysis (equal); Investigation (equal). **You Li:** Data curation (supporting); Formal analysis (supporting). **Hui Che:** Data curation (supporting); Formal analysis (supporting). **Ying Chen:** Data curation (supporting); Formal analysis (supporting). **Jianghui Dong:** Writing‐original draft (equal); Writing‐review & editing (equal). **Cory Xian:** Writing‐original draft (equal); Writing‐review & editing (equal). **Dengshun Miao:** Writing‐original draft (supporting). **Liping Wang:** Supervision (equal); Writing‐original draft (lead); Writing‐review & editing (lead). **Yongxin Ren:** Funding acquisition (lead); Project administration (lead); Supervision (equal); Writing‐original draft (equal); Writing‐review & editing (equal).

## Data Availability

The data that support the findings of this study are available from the corresponding author upon reasonable request.
